# A multimodal approach for assessing the risk of cervical spine injury in low-speed rear-end collisions: kinematic and clinical responses

**DOI:** 10.3389/fbioe.2026.1743163

**Published:** 2026-03-06

**Authors:** Hee Young Lee, Kang Hyun Lee, Oh Hyun Kim, Hyunjung Kim, Chan Young Kang, Guan Hee Kim, Nam Hyung Kim, Hyun Youk

**Affiliations:** 1 Center for Automotive Medical Science Institute, Yonsei University Wonju College of Medicine, Wonju, Gangwon, Republic of Korea; 2 Department of Radiology, Wonju Severance Christian Hospital, Wonju, Gangwon, Republic of Korea; 3 Korea Automobile Insurance Repair & Training Center, Korea Insurance Development Institute, Icheon, Republic of Korea

**Keywords:** cervical spine injury, electromyogram (EMG), minor motor vehicle crashes, magnetic resonance imaging (MRI), nerve conduction velocity (NCV)

## Abstract

**Purpose:**

While advancements in vehicle safety have reduced injury severity in low-speed collisions, concerns about excessive treatment costs and the social implications of minor collisions persist. Cervical spine injuries, in particular, remain challenging to assess despite minimal vehicle damage. This study aimed to establish objective criteria for evaluating cervical spine injury risk in low-speed rear-end collisions and determine whether such injuries occur at impact velocities up to 8 km/h.

**Methods:**

Sixteen volunteers underwent controlled rear-end collisions using passenger vehicles and bumper cars at impact velocities ranging from 1.54 to 8.86 km/h. Kinematic parameters—including velocity change (ΔV), peak acceleration, and neck injury criteria (NIC)—were recorded. Cervical MRI scans were obtained pre- and post-collision, while electromyography (EMG) and nerve conduction studies (NCS) assessed neuromuscular function. Pain questionnaires were administered immediately after the test and 1 week later.

**Results:**

Impact velocity, offset percentage, and collision type significantly influenced acceleration forces and NIC values, with passenger vehicle collisions generating higher forces than bumper car collisions. Post-collision MRI scans showed no structural cervical spine damage, and no EMG or NCS abnormalities were detected. While 58.3% of participants reported immediate post-collision pain, this decreased to 25% within a week, primarily affecting the cervical spine and lower back.

**Conclusion:**

The risk of cervical spine injury in low-speed rear-end collisions (ΔV≤8 km/h) appears minimal, with no significant structural or neuromuscular abnormalities observed. Further research is needed to assess long-term effects and improve occupant comfort.

## 1 Introduction

Low-speed rear-end collisions are a common cause of whiplash-associated disorder (WAD), a term used to describe a range of injuries to the neck caused by or related to sudden distortion of the neck, often as a result of rear-end vehicle accidents (Haldorsen et al., 2003). Whiplash injuries frequently occur, even in cases where vehicle deformation is minimal, such as minor dents or scrapes. Whiplash injury is an acceleration-deceleration mechanism of energy transmitted to the neck (Spitzer et al., 1995). An analysis of car accidents in Germany from 1990 revealed that in approximately 94% of rear-end collisions involving reported injuries, at least one passenger complained of a whiplash injury (Fahrzeugsicherheit 90, 1994). In 65% of all injury cases, the change in velocity (ΔV) due to collision did not exceed 15 km/h (Eichberger, 1995). Interestingly, the incidence of reported whiplash injuries decreased in proportion to the extent of the vehicle deformation.

Whiplash-associated disorder (WAD) encompasses a heterogeneous spectrum of clinical manifestations, ranging from subjective symptoms such as neck pain, stiffness, and headache to objectively verifiable structural or neurological injuries. From a pathophysiological perspective, WAD may involve soft tissue strain, ligamentous microtrauma, intervertebral disc stress, or neural element involvement resulting from rapid acceleration–deceleration forces applied to the cervical spine. Importantly, not all components of WAD are equally detectable using current diagnostic modalities. Magnetic resonance imaging (MRI) is sensitive to macroscopic structural abnormalities, including fractures, disc herniation, spinal cord injury, and major soft tissue lesions, but has limited ability to identify microscopic tissue damage or pain-related functional disturbances. Similarly, electromyography (EMG) and nerve conduction studies (NCS) can detect electrophysiological abnormalities associated with nerve root or peripheral nerve dysfunction, but may remain normal in cases of isolated muscle strain or pain without neural involvement. In the present study, “injury” was operationally defined as the presence of objectively detectable structural abnormalities on MRI or electrophysiological abnormalities on EMG/NCS following low-speed rear-end collisions. Subjective pain symptoms were evaluated separately and were not considered sufficient evidence of injury in the absence of corresponding objective findings.

There have been several studies aimed at reducing social costs to address these issues. Castro et al. presented that it conducted an experiment with the effective collision speed in the range of 8.3–14.2 km/h on volunteers and reported that the hyperextension of the neck did not occur. They concluded that the “limit of harmlessness” from rear-end collisions is 10–15 km/h (Castro et al., 1997). Brault et al. showed that the possibility of neck injury varied depending on the headrest position (Brault et al., 1998). Szabo et al. presented the possibility of injury when an additional impact was applied to patients with past illnesses (Szabo et al., 1994). Even in Korea, social issues regarding excessive medical expenses are continuously being raised, even though only damage to the bumper of a compact car occurs in a minor collision. Therefore, several studies have been conducted on vehicle kinematics and neck injuries in low-speed collision accidents. Park et al. conducted a laboratory experiment targeting 50 Korean adult men in their 30s–50 s, assuming a rear-end collision with an effective collision speed (ΔV) of 5–8 km/h. In this experiment, 44 out of 50 subjects were not found to have any abnormal symptoms, and none of the remaining 6 subjects complained of neck discomfort (Park et al., 2013). Stiffness and damping coefficients have also been introduced to predict the acceleration (impact pulse) based on test results and vehicle kinematics (Han and Park, 2001; Han, 2007). A study was also conducted to compare accident analysis and prediction simulation tools developed based on crash dynamics with the actual crash test results (Cipriani et al., 2002). In addition, research was conducted on the accuracy of the information extracted from the accident recording device by comparing the information recorded in the accident recording device (EDR) mounted on the vehicle with the data obtained from the acceleration sensor during the crash test (Wilkinson et al., 2013; Xing et al., 2016).

Nonetheless, the biomechanical threshold for neck strain in mild MVCs remains controversial. While several experimental and epidemiological studies have proposed so-called “harmless” speed thresholds based on collision kinematics, clinical observations continue to report a substantial prevalence of neck-related symptoms even after minor rear-end impacts. This discrepancy highlights an ongoing debate between symptom-based clinical outcomes and biomechanically defined injury thresholds. Moreover, mild whiplash-associated disorders are often reported to be difficult to verify using conventional diagnostic tools, including MRI and CT, particularly in the absence of overt neurological deficits. Importantly, most prior studies have evaluated either biomechanical parameters, imaging findings, or clinical symptoms in isolation. Therefore, the present study aimed to address this gap by simultaneously integrating kinematic analysis with advanced radiological imaging (MRI) and electrodiagnostic assessments (EMG and nerve conduction studies) in a controlled low-speed rear-end collision model. By combining these complementary modalities, we sought to provide a more comprehensive and objective evaluation of cervical spine injury risk in minor rear-end collisions.

## 2 Methods

### 2.1 Participants

A total of 16 adult volunteers (eight males and eight females) participated in this study. Participant age and sex were recorded at enrolment. Detailed anthropometric data, including height, weight, and body mass index (BMI), as well as detailed information regarding prior cervical spine disease or driving and seat usage habits, were not systematically collected. All participants were required to be capable of driving and underwent a pre-test medical assessment to confirm the absence of acute cervical spine pathology at the time of participation. All participants provided written informed consent before participating in the study. This clinical study complied with the International Conference on Harmonization (ICH) Guidelines and the principles of the Declaration of Helsinki. This study was approved by the research ethics committee of Wonju Severance Christian Hospital, Yonsei University (IRB approval no.: CR320057) and the Clinical Research Information Service of Korea Disease Control and Prevention Agency (Trial registration no.: KCT0005769).

### 2.2 Rear-end collision tests in low-speed range

Objective impact data were collected from low-speed rear-end collision simulations, followed by medical evaluation by emergency medicine, rehabilitation, and radiology specialists. One compact passenger vehicle (Spark, M2JC) and one sport utility vehicle (Sorento, UM) were used to examine the effects of collision type on vehicle kinematics and occupant responses. All collision tests were conducted using production vehicles equipped with factory-installed seats, head restraints, seatbelts, and energy-absorbing structures, without any aftermarket modifications. Participants wore standard three-point seatbelts, which were fastened according to manufacturer specifications. Seatbelt restraint force and pretension characteristics were not independently controlled or measured, as these parameters are integrated into the vehicle safety system. Prior to testing, participants adjusted the seat position and head restraint to achieve a normal driving posture based on individual comfort and safety. Seat parameters, including headrest height and backrest angle, were not mechanically fixed or quantitatively standardized, in order to reflect typical real-world driving conditions. This approach was applied consistently across all test scenarios and vehicle types. The collision speed, delta-V, maximum acceleration, average acceleration, and Neck Injury Criterion (NIC) values were collected by attaching acceleration sensors to the vehicle’s tunnel rear, B-pillar, passenger’s head, and T1, and a camera was installed to record the collision scene. (Table 1).

**TABLE 1:** Overview of real vehicle low-speed collision accident reproduction test.

Indicators	Passenger car		SUV	
Model	Spark (M2JC)		Sorento (UM)	
Curb-weight (kg)	1,110		1,975	
Product year (YYYY)	2018		2018	
Photo	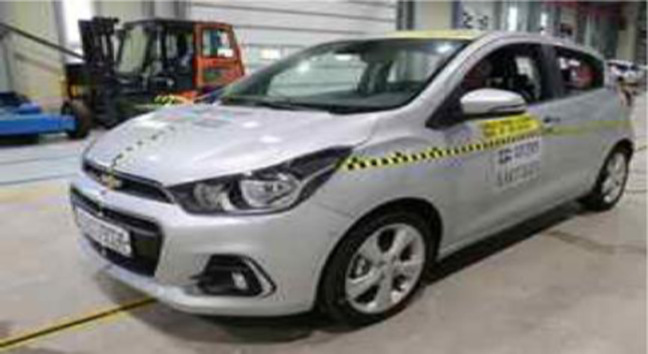		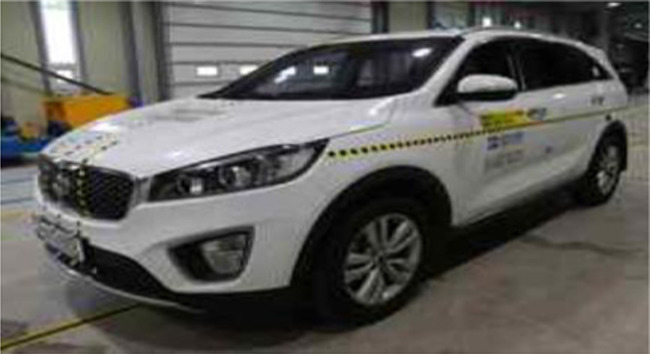	
Instrument	Accelerometer	Participant		Camera
		Male	Female	
	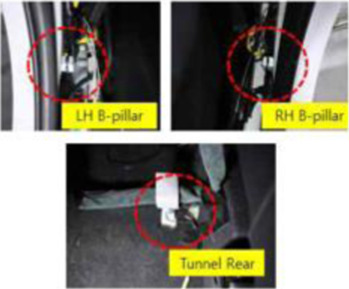	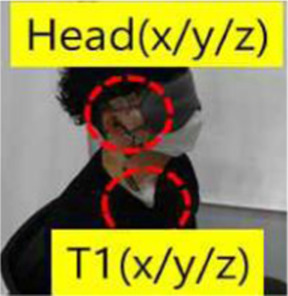	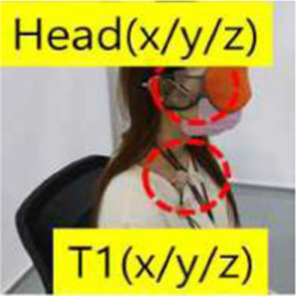	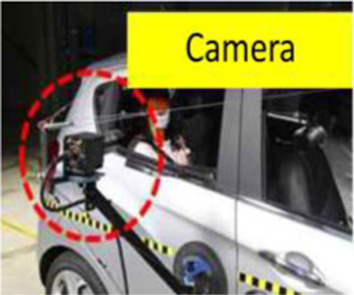

None.

Abbreviations: SUV, sport utility vehicle.

### 2.3 Rear impact tests with bumper cars

**FIGURE 1:**
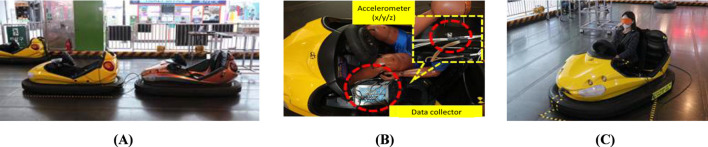
Overview of bumper car crash test. **(A)** Commercially operated vehicle-type bumper car used for rear-impact testing (Max speed 11 ± 1 km/h) **(B)** Instrumentation setup. Tri-axial acceleration sensors were installed at the vehicle rear tunnel and B-pillar to capture vehicle kinematic responses, and at the participant’s head and T1 vertebral level to record occupant kinematics. **(C)** Test participant seated in the bumper car with standard seating posture and head position during the rear-impact test.

Bumper car impact tests were included as a comparative low-speed exposure reference rather than as a biomechanically equivalent surrogate for passenger vehicle rear-end collisions (Figure 1). The purpose of this comparator was to contextualize cervical loading under familiar low-speed impact conditions. A vehicle-type bumper car with a mass of 215 kg, operating on an electrically embedded floor system, was used, allowing controlled speeds ranging from 7 to 11 km/h. Collision tests were conducted with one male and one female participant alternately seated in the colliding and struck bumper cars. Quantitative impact parameters, including collision speed, change in velocity (ΔV), peak acceleration, mean acceleration, and Neck Injury Criterion (NIC), were recorded using an acceleration sensor and a data acquisition system installed within the bumper car (Figure 1). The bumper car impacts produced ΔV values less than 8 km/h, overlapping with the lower range of passenger vehicle collision tests. This overlap enabled a contextual comparison of low-level acceleration exposure and cervical loading magnitude across impact conditions, while acknowledging inherent differences in vehicle mass, structural stiffness, seating configuration, and impact pulse characteristics.

### 2.4 Impact scenarios

In Table 2, collision direction was defined using a clock-face–based representation referenced to the longitudinal axis of the struck vehicle. A “06:00” direction indicated a purely longitudinal rear-end impact aligned with the vehicle centerline (0°), whereas “05:30” represented an oblique rear-end impact corresponding to a 30° deviation from the longitudinal axis in the clockwise direction. Offset was defined as the percentage of lateral overlap between the front bumper of the colliding vehicle and the rear bumper of the struck vehicle, calculated relative to the total bumper width of the struck vehicle. For example, a 40% offset indicated that 40% of the bumper widths overlapped at the point of impact.

**TABLE 2:** Collision test scenarios including vehicle to vehicle and bumper car to bumper car.

Test no.	Vehicle model		Speed (km/h)	Offset (%)	Direction (clockwise: Evaluation (∘))	Participant no. (Sex/Age)	
						FD	RR
	Colliding	Collided					
V1	Sorento	Spark	8	100	06:00	P1 (M/27)	P2 (F/33)
V2	Sorento	Spark	8	100	06:00	P3 (M/27)	P4 (F/25)
V3	Spark	Sorento	8	40	05:30	P5 (M/39)	P6 (F/29)
V4	Spark	Sorento	8	40	05:30	P7 (F/35)	P8 (M/38)
V5	Spark	Sorento	9.5	15	06:00	P9 (M/45)	P10 (F/46)
V6	Spark	Sorento	9.5	15	06:00	P11 (F/46)	P12 (M/42)
B1	Bumper car	Bumper car	8	100	06:00	P13 (M/27)	N/A
B2	Bumper car	Bumper car	8	100	06:00	P14 (F/27)	N/A
B3	Bumper car	Bumper car	8	40	05:30	P15 (M/25)	N/A
B4	Bumper car	Bumper car	8	40	05:30	P16 (F/31)	N/A

Participants riding the bumper cars sat only in the front driver’s seat. Collision direction is expressed using a clock-face–based notation referenced to the struck vehicle’s longitudinal axis (06:00 = 0°, 05:30 = 30° oblique rear-end). Offset represents the percentage of lateral bumper overlap relative to the struck vehicle’s bumper width.

Abbreviations: V, vehicle to vehicle; B, bumper car to bumper car; FD, front driver seat; RR, rear right seat; P, participant; M, male; F, female.

### 2.5 Medical examination

We performed cervical MRI before the crash test and conducted an MRI after the test. Follow-up MRI was performed within 72 h after the crash test. A 3-T MR imager (Magnetom Skyra; Siemens Healthcare, Erlangen, Germany) with a dedicated spine surface coil was used. The examinations included axial T2-weighted turbo spin-echo (TSE), axial gradient-echo, and sagittal T2-weighted TSE. Cervical spine radiography and MR images were retrospectively evaluated by a musculoskeletal radiologist with 3 years of experience, who was blinded to the patient information. The analyzed MR findings included the presence of fracture, spinal cord edema or hemorrhage, prevertebral fluid collection, tear of the paraspinal muscle or ligament, and alignment changes such as straightening. The low signal intensity line newly observed in the cervical spine on sagittal T2-weighted MRI images obtained after the crash test was interpreted as a fracture. Similarly, increased signal intensity was noted on both axial T2-weighted images and sagittal T2-weighted images of the spinal cord, which were interpreted as indicative of acute spinal cord injury. Dark signal intensity on axial gradient-echo images of the spinal cord was interpreted as indicative of cord hemorrhage. Electromyography (EMG) and nerve conduction studies (NCS) were performed 2 weeks after the crash test. This timing was selected to allow potential electrophysiological abnormalities associated with nerve root irritation, peripheral nerve dysfunction, or muscle injury to become detectable beyond transient acute physiological responses. Immediate post-impact testing was avoided to reduce the influence of short-lived neuromuscular activation or fatigue that may not reflect clinically meaningful injury. Rehabilitation specialists meticulously reviewed electrodiagnostic reports and analyzed key neurophysiological parameters such as nerve conduction velocity, compound muscle action potential (CMAP), sensory nerve action potential (SNAP), and EMG findings to evaluate neuromuscular function in the cervical region.

### 2.6 Pain questionnaire

After the crash test, participants were asked to report whether pain had occurred and to indicate the anatomical location of any pain experienced. Pain assessments were conducted immediately after the crash test and again 1 week later. Participants specified the presence of pain in the cervical spine (neck), shoulders and upper arms, back, lumbar spine (lower back), or waist. Quantitative pain intensity scales, such as the Visual Analogue Scale or numerical rating scales, were not employed, as pain severity was not a primary outcome measure of this study.

## 3 Results

### 3.1 Rear-end collision tests

Table 3 presents the results of the low-speed rear-end collision tests, including the changes in velocity (ΔV), maximum acceleration, and mean acceleration recorded at two key points: the vehicle body tunnel (x/y/z, three axes) and the B-pillar left/right (x/y/z, each of the three axes). The data were processed using a CFC60 filter, as recommended by SAE J211 (SAE International, 2022), and analyzed according to the ISO/TR 12353–3:2013(E) technical report (International Organization for Standardization, 2013). The tests involved collisions between colliding and collided vehicles, as well as bumper cars, with variations in the impact speed, offset percentage, and collision direction. The ΔV ranged from 1.54 km/h to 7.46 km/h for passenger vehicle collisions and 5.76 km/h to 6.63 km/h for bumper car collisions. Maximum acceleration (max. Acc.) recorded in passenger vehicle collisions ranged from 0.38 g to 4.05 g, while bumper car collisions exhibited lower acceleration values between 1.17 g and 1.40 g. Similarly, the mean acceleration (Mean Acc.) for passenger vehicle collisions varied between 0.20 g and 1.71 g, whereas bumper car collisions showed values between 0.57 g and 0.76 g. The neck injury criteria (NIC), an indicator of potential neck strain, differed across test conditions. The highest NIC value (5.0 m^2^/s^2^) was recorded in a collision with an impact speed of 8.861 km/h and a 40% offset, whereas the lowest NIC (0.5 m^2^/s^2^) was observed in a collision with an impact speed of 9.566 km/h and a 15% offset. Notably, the NIC values in bumper car collisions remained below 2.0 m^2^/s^2^, suggesting a lower risk of neck strain compared to passenger vehicle collisions. Additionally, the front driver (FD) and rear right (RR) acceleration values varied depending on the impact conditions. These findings demonstrate condition-dependent differences in acceleration measures and NIC values according to impact velocity, offset percentage, and collision type, based on descriptive comparisons across the tested scenarios. Passenger-vehicle collisions generally exhibited higher acceleration and NIC values than bumper-car collisions in the tested scenarios, reflecting descriptive differences in biomechanical response between collision types.

**TABLE 3:** Results of low-speed collision test with occupants.

Test no.	Vehicle model		Speed (km/h)	Offset, direction (%, Clockwise)	Delta V (km/h)	Max. Acc. (g)	Mean Acc. (g)	NIC (m^2^/s^2^)	
	Collided	Colliding						FD	RR
V1	Spark	Sorento	8.577	100, 06	7.46	2.95	1.64	2.8	5.1
V2	Spark	Sorento	8.577	100, 06	7.33	3.04	1.71	3.7	1.4
V3	Sorento	Spark	8.861	40, 05	3.51	3.51	0.79	2.0	3.3
V4	Sorento	Spark	8.861	40, 05	3.64	4.05	0.77	5.0	3.4
V5	Sorento	Spark	9.566	15, 06	1.67	0.51	0.25	2.8	2.0
V6	Sorento	Spark	9.566	15, 06	1.54	0.38	0.20	0.5	0.5
B1	Bumper car	Bumper car	7.276	100, 06	5.76	1.17	0.66	1.1	​
B2	Bumper car	Bumper car	7.376	100, 06	6.37	1.40	0.73	1.6	
B3	Bumper car	Bumper car	7.234	40, 05	6.63	1.33	0.57	1.1	
B4	Bumper car	Bumper car	7.247	40, 05	5.85	1.39	0.76	2.0	

None.

Abbreviations: V, vehicle to vehicle; B, bumper car to bumper car; △V, change of velocity; ACC., acceleration; Max., maximum; NIC, neck injury criteria; FD, front driver; RR, rear right.

### 3.2 Pain questionnaire

Table 4 summarizes the responses to the pain questionnaire administered immediately after the crash test and again 1 week later. Among the 12 vehicle occupants, seven (58.3%) reported experiencing pain immediately following the collision, whereas none of the four bumper car occupants reported any pain. The most commonly reported pain locations were the cervical spine (5 participants, 71.4%), followed by the lower back (3 participants, 42.9%), shoulders and upper extremities (2 participants, 28.6%), and lumbar spine (1 participant, 14.3%). One week after the test, three of the 12 vehicle occupants (25.0%) continued to report pain, primarily in the cervical spine (2 participants, 66.7%) and lower back (2 participants, 66.7%).

**TABLE 4:** Results of a questionnaire on whether participants felt pain after the crash test.

Time of survey	Body region with pain	No. of participants reporting pain	No. of participants with no pain
Immediately after crash test	Cervical spine	5	9
	Shoulder and upper arm	2	
	Lumbar spine	1	
	Waist	3	
One week after crash test	Cervical spine	2	13
	Shoulder and upper arm	0	
	Lumbar spine	0	
	Waist	2	

Available responding to multiple pain body regions.

Abbreviations: None.

### 3.3 MRI readings before and after collision test

Table 5 presents the MRI findings before and after the crash test. Prior to the test, all participants exhibited no abnormal signal intensities in the bone or soft tissue on cervical MRI scans. In terms of cervical spine alignment, 15 of 16 participants exhibited a straight alignment, while 1 displayed a normal curvilinear alignment. This baseline straightening was interpreted as a postural or physiological variant rather than a pathological finding, as cervical alignment can vary depending on positioning and muscle tone during imaging. Post-collision MRI showed no fractures, no changes in signal intensity, and no abnormalities in the cervical spine, spinal cord, or surrounding soft tissues. Additionally, cervical spine alignment remained unchanged in all participants. Importantly, no interval changes were identified on pre-to post-impact comparisons, supporting the conclusion that low-speed rear-end collisions did not induce new structural damage to the cervical spine or surrounding tissues.

**TABLE 5:** Cervical MRI findings before and after the collision test in all participants.

Participant	Fracture		Spinal cord edema/Hemorrhage		Prevertebral fluid collection		Paraspinal muscle or ligament edema		Straightening	
	Before	After	Before	After	Before	After	Before	After	Before	After
P1	-	-	-	-	-	-	-	-	-	-
P2	-	-	-	-	-	-	-	-	+	+
P3	-	-	-	-	-	-	-	-	+	+
P4	-	-	-	-	-	-	-	-	+	+
P5	-	-	-	-	-	-	-	-	+	+
P6	-	-	-	-	-	-	-	-	+	+
P7	-	-	-	-	-	-	-	-	+	+
P8	-	-	-	-	-	-	-	-	+	+
P9	-	-	-	-	-	-	-	-	+	+
P10	-	-	-	-	-	-	-	-	+	+
P11	-	-	-	-	-	-	-	-	+	+
P12	-	-	-	-	-	-	-	-	+	+
P13	-	-	-	-	-	-	-	-	+	+
P14	-	-	-	-	-	-	-	-	+	+
P15	-	-	-	-	-	-	-	-	+	+
P16	-	-	-	-	-	-	-	-	+	+

Cervical MRI was performed both before and after the collision test in all participants. Images were retrospectively evaluated by a musculoskeletal radiologist who was blinded to participant information.

Abbreviations: (+), positive finding; (−), negative finding.

### 3.4 EMG/NCS analysis before and after collision test

Table 6 shows the motor and sensory nerve conduction study results for the different nerve segments tested.

**TABLE 6:** Electromyography (EMG) results for nerve conduction studies after collision test.

Nerve conduction parameters	Left side	Right side	t	p-value
Median motor (Wrist-APB)				
Latency (ms)	2.90 ± 0.30	2.96 ± 0.39	−0.852	0.408
Amplitude (mV, onset)	12.03 ± 1.87	12.71 ± 1.78	−1.522	0.149
Amplitude (mV, peak)	19.43 ± 3.62	20.59 ± 2.94	−1.240	0.234
Stimulus intensity (mA)	21.19 ± 9.39	21.89 ± 13.59	−0.235	0.817
Duration (ms)	5.84 ± 0.69	5.65 ± 0.55	1.213	0.244
Area (ms*mV)	39.16 ± 8.00	40.31 ± 6.75	−0.686	0.503
Median motor (elbow-wrist)				
Latency (ms)	6.81 ± 0.61	6.89 ± 0.58	−0.694	0.498
Amplitude (mV, onset)	11.48 ± 2.06	11.48 ± 2.12	0.000	1.000
Amplitude (mV, peak)	18.63 ± 3.06	18.53 ± 3.34	0.131	0.898
Stimulus intensity (mA)	38.59 ± 25.67	39.27 ± 20.11	−0.134	0.896
Duration (ms)	5.93 ± 0.74	5.73 ± 0.63	1.611	0.128
Area (ms*mV)	37.13 ± 7.31	36.66 ± 6.87	0.348	0.733
Ulnar motor (Wrist-APB)				
Latency (ms)	2.47 ± 0.33	2.60 ± 0.38	−1.864	0.082
Amplitude (mV, onset)	10.56 ± 1.40	10.72 ± 1.91	−0.483	0.636
Amplitude (mV, peak)	18.17 ± 2.47	18.69 ± 3.29	−0.860	0.403
Stimulus intensity (mA)	16.96 ± 6.19	19.69 ± 7.95	−1.209	0.245
Duration (ms)	5.84 ± 0.58	5.71 ± 0.45	1.698	0.110
Area (ms*mV)	35.98 ± 4.91	34.99 ± 6.12	0.972	0.347
Ulnar motor (bl. Elbow-wrist)				
Latency (ms)	6.47 ± 0.59	6.44 ± 0.51	0.284	0.780
Amplitude (mV, onset)	9.68 ± 1.64	9.70 ± 2.11	−0.039	0.970
Amplitude (mV, peak)	17.18 ± 2.84	17.00 ± 3.82	0.203	0.842
Stimulus intensity (mA)	26.59 ± 18.96	28.22 ± 14.18	−0.357	0.726
Duration (ms)	6.08 ± 0.57	6.01 ± 0.56	0.541	0.525
Area (ms*mV)	33.83 ± 5.34	32.20 ± 7.03	1.118	0.281
Median sensory (finger-wrist)				
Latency (onset) (ms)	2.19 ± 0.26	2.24 ± 0.22	−1.191	0.252
Latency (peak) (ms)	3.04 ± 0.32	3.02 ± 0.32	0.333	0.744
Amplitude (mV)	73.69 ± 33.20	71.25 ± 25.65	0.697	0.497
Stimulus intensity (mA)	8.18 ± 2.85	7.61 ± 2.43	1.257	0.228
Ulnar sensory (wrist-finger)				
Latency (onset) (ms)	2.09 ± 0.34	2.09 ± 0.36	−0.008	0.993
Latency (peak) (ms)	2.93 ± 0.37	2.87 ± 0.39	0.903	0.381
Amplitude (mV)	48.58 ± 24.38	49.87 ± 22.21	−0.446	0.662
Stimulus intensity (mA)	6.28 ± 2.45	6.44 ± 2.55	−0.514	0.615

Rehabilitation specialists meticulously reviewed electrodiagnostic reports and analyzed key neurophysiological parameters.

Abbreviations: APB, abductor pollicis brevis; BI., below.

For the Median Motor (Wrist-APB) nerve conduction, latency, amplitude, stimulus intensity, duration, and area values showed minimal variation between the left and right sides. The latency was 2.90 ± 0.30 ms on the left and 2.96 ± 0.39 ms on the right (p = 0.408). Similarly, the peak amplitude was 19.43 ± 3.62 mV on the left and 20.59 ± 2.94 mV on the right (p = 0.234). None of these differences were statistically significant.

In the Median Motor (Elbow-Wrist) segment, latency values (6.81 ± 0.61 ms for the left and 6.89 ± 0.58 ms for the right) were nearly identical (p = 0.498). The peak amplitude and other parameters also showed negligible differences with no statistically significant variations. For the Ulnar Motor (Wrist-APB) conduction, latency on the left side was 2.47 ± 0.33 ms and 2.60 ± 0.38 ms on the right (p = 0.082), showing a trend toward difference but not reaching statistical significance. Amplitude and stimulus intensity also showed minor variations, but all p-values exceeded 0.05. Similarly, the Ulnar Motor (Bl. Elbow-Wrist) segment, no significant differences were observed in latency, amplitude, or other conduction parameters.

Regarding sensory nerve conduction, the Median Sensory (Finger-Wrist) latency (onset) was 2.19 ± 0.26 ms on the left and 2.24 ± 0.22 ms on the right (p = 0.252), with peak latency and amplitude values remaining statistically comparable. The Ulnar Sensory (Wrist-Finger) conduction also showed no significant differences between the left and right sides, with latency (onset) of 2.09 ± 0.34 ms on the left and 2.09 ± 0.36 ms on the right (p = 0.993). Overall, the NCS results indicated normal findings, with symmetrical motor and sensory nerve functions on both the left and right sides. The CMAP and SNAP studies showed no abnormalities, and no statistically significant differences were observed between the left and right sides in any of the measured parameters. Additionally, electromyography (EMG) results revealed no abnormal spontaneous activity at rest in the examined muscles, with normal motor unit potentials observed during volitional contraction. During maximal effort, a partial-to-complete interference pattern was observed in all muscles. No signs of cervical radiculopathy or peripheral polyneuropathy were noted. These findings suggested that there were no electrophysiological abnormalities in the tested population.

## 4 Discussion

This study aimed to investigate the effects of low-speed rear-end collisions on cervical spine injury risk and assess the electrophysiological changes in motor and sensory nerve conduction following such collisions. Our findings, based on rear-end collision tests, pain questionnaires, MRI scans, and electromyography (EMG) and nerve conduction study (NCS) analyses, provide critical insights into the biomechanical and neurophysiological effects of low-speed crashes on vehicle occupants. The results indicated that while the biomechanical responses varied according to the collision type, no significant electrophysiological abnormalities or structural damage were observed following the rear-end collision tests.

### 4.1 Biomechanical response and risk of neck injury

The impact speed, offset ratio, and collision type significantly influence neck acceleration and the risk of neck injuries in minor collisions, particularly in rear-end impacts. Our findings align with previous research, reinforcing that variations in these parameters contribute to different biomechanical responses and injury risks. Higher impact speeds generally correlate with increased neck injury risk. In our study, ΔV ranged from 1.54 km/h to 7.46 km/h for passenger vehicle collisions and 5.76 km/h to 6.63 km/h for bumper car collisions, with corresponding variations in acceleration and NIC values. Notably, the highest NIC value (5.0 m^2^/s^2^) was recorded at an impact speed of 8.861 km/h with a 40% offset. These results support prior research indicating that even low-speed collisions can generate significant neck forces (Jo and Kim, 2007). The offset ratio plays a crucial role in force distribution during collisions. Our results showed that a 40% offset produced the highest NIC value (5.0 m^2^/s^2^), while a 15% offset resulted in the lowest NIC value (0.5 m^2^/s^2^). This suggests that greater offset misalignment may lead to increased neck strain, as supported by previous studies indicating that oblique impacts generate higher reaction forces on the neck (Ono et al., 2009). Different collision types produce distinct biomechanical responses. Passenger vehicle collisions exhibited higher maximum acceleration (up to 4.05 g) and NIC values compared to bumper car collisions, where NIC remained below 2.0 m^2^/s^2^. These findings indicate that bumper car collisions impose lower neck injury risks, likely due to their structural design and energy absorption characteristics. This aligns with previous research showing that impact type influences cervical spine strain (Perry et al., 2019). From a biomechanical perspective, the cervical spine is particularly vulnerable to inertial loading during rear-end collisions due to its segmented anatomy, relatively low intrinsic muscular support, and the mass of the head. During low-speed rear impacts, the torso is accelerated forward by the seatback, while the head initially lags behind because of inertia, producing relative motion between the head and upper thorax. This transient phase of cervical extension, followed by flexion, is characteristic of whiplash-like motion. Segmentally, this process involves non-uniform motion across cervical levels, with differential displacement and shear forces occurring particularly in the mid-to-lower cervical spine. Even in the absence of detectable structural injury, such rapid deformation and shear loading may contribute to soft tissue strain or transient neuromuscular responses. Therefore, displacement-based metrics such as the Neck Injury Criterion (NIC) provide important insight into cervical spine loading mechanisms, even in collision scenarios traditionally considered minor. While impact parameters significantly affect neck injury risk, individual factors such as occupant posture, seat design, and vehicle characteristics must also be considered for a comprehensive injury risk assessment. Further studies incorporating a wider range of collision scenarios and occupant variables could enhance our understanding of neck injury mechanisms in low-speed impacts.

### 4.2 Radiological response: MRI findings

Our study demonstrated that low-speed rear-end collisions did not result in structural damage to the cervical spine or surrounding soft tissues, as confirmed by MRI findings. These results contrast with prior research suggesting that minor collisions can lead to cervical injuries, including ligamentous damage and spinal cord involvement. MRI is often used to assess soft tissue injuries following trauma. However, in our study, no fractures, abnormal signal intensities, or soft tissue changes were observed before or after the collision test. This supports existing literature indicating that MRI has limited utility in detecting clinically significant cervical spine injuries in patients with normal CT scans and no neurological deficits (Foster et al., 2022). Although previous reports have described rare cases of cervical spinal cord injury following low-impact trauma, sometimes resulting in severe complications such as cardiorespiratory arrest (Mayà-Casalprim et al., 2020), our results suggest that controlled low-speed rear-end collisions do not produce detectable structural cervical spine injuries in healthy adult volunteers. This discrepancy highlights the substantial variability in injury outcomes and suggests that individual anatomical differences, pre-existing cervical conditions, and uncontrolled real-world factors may play a more prominent role in such cases. Given the cost, limited specificity, and potential for overdiagnosis, routine MRI evaluation after low-speed rear-end collisions may not be warranted in the absence of neurological symptoms or abnormal CT findings (Malhotra et al., 2023). These findings reinforce the importance of individualized clinical assessment rather than routine imaging in minor collision scenarios.

### 4.3 Electrophysiological responses: nerve conduction and EMG

Our findings suggest that low-speed rear-end collisions do not induce any measurable electrophysiological abnormalities, as evidenced by normal EMG and NCS results. In our study, the absence of significant differences in nerve conduction or muscle activity between the left and right sides, as well as the normal motor unit potentials observed during volitional contraction, implies that low-speed impacts did not cause detectable neurological damage. Although no significant changes in EMG were detected post-collision, research indicates that cervical muscles experience substantial activation during low-speed rear impacts (Marianne et al., 2019). These muscle responses may play a key role in the injury mechanisms, even when no clear structural damage is present. Our study did not identify spontaneous muscle activity at rest or abnormal patterns during contraction, suggesting that while muscle activation occurs, it does not necessarily lead to long-term damage or dysfunction in this context. Studies involving electrophysiological assessments post-vertebral trauma have demonstrated that even minor spinal injuries may affect neural pathways, which can remain subclinical initially (Il’yasevich et al., 2009). However, in our cohort, no signs of cervical radiculopathy or peripheral polyneuropathy were detected. The lack of abnormal EMG activity and the symmetrical nerve conduction across both sides suggest that, in this controlled low-impact collision setting, the cervical spine and associated neural pathways were not adversely affected. Several limitations related to the electrophysiological assessment should be acknowledged. Pre-impact baseline EMG/NCS measurements were not obtained; therefore, post-impact findings could not be directly compared with individual baseline values. As a result, the analysis relied primarily on established normal reference ranges and left–right symmetry comparisons. While side-to-side symmetry is commonly used in clinical electrodiagnostic practice, this approach may not detect subtle bilateral or subclinical changes in neuromuscular function. In assessing the potential for cervical spine injury from minor collisions, it is important to note that factors such as the deceleration impact and muscle strain may not directly correlate with injury severity in all cases. Another previous study have found no strong correlation between the change in velocity during real-life collisions and cervical spine injury severity, suggesting that other variables, such as the position of the head and neck, may contribute to injury risk (Elbel et al., 2009). In addition, electrophysiological changes occurring in the acute phase immediately after impact or within the first few days may not have been captured. Transient alterations in muscle activation or nerve excitability that resolve within this period could therefore be underestimated. Consequently, the absence of abnormal EMG/NCS findings at 2 weeks does not exclude the possibility of short-lived acute neuromuscular responses following low-speed rear-end collisions. This finding highlights the need for further research to explore the potential for undetected cervical spine damage in minor collisions, particularly focusing on the relationship between electrophysiological changes and clinical outcomes. Further studies with larger sample sizes and varying impact conditions are needed to refine our understanding of how minor collisions impact the cervical spine and associated neurological structures.

### 4.4 Discrepancies with previous studies and interpretation of “harmless” speed thresholds

Several peer-reviewed studies have highlighted limitations in using ΔV alone as a predictor of cervical injury risk. For example, Schmitt et al. reported that ΔV was not a conclusive predictor of cervical spine injury in real-world motor vehicle crashes, with low correlations between ΔV and clinical symptom severity or disability scores, suggesting that crash severity metrics beyond ΔV are needed to assess injury outcomes (Elbel et al., 2009). A comprehensive review comparing volunteer rear-end impact studies with real-world crash outcomes found that neck symptom frequency was similar between controlled volunteer protocols and actual crashes, indicating that volunteer studies can model aspects of injury risk but that ΔV thresholds should not be generalized across contexts (Cormier et al., 2018). In addition, analysis of minor rear crashes in the United States showed that a substantial proportion of occupants involved in collisions with ΔV ≤15 km/h still reported neck complaints, further questioning the existence of a universal harmless speed threshold (Bartsch et al., 2008).

### 4.5 Study limitations

This study has several limitations that should be acknowledged. First, the sample size was relatively small and consisted exclusively of healthy adult volunteers aged 25–46 years, which limits the external validity of the findings. The results may not be generalizable to older adults, individuals with osteoporosis or degenerative cervical spine changes, or other vulnerable populations who may exhibit different biomechanical and physiological responses to low-speed rear-end collisions. Special populations, including elderly individuals, obese occupants, and those with pre-existing cervical spine conditions, were not included. Second, only basic demographic information, such as age and sex, was recorded. Detailed anthropometric data, including height, weight, body mass index, prior cervical spine medical history, and habitual seat usage, were not collected. These factors may influence individual susceptibility to cervical spine injury and could not be evaluated in the present study. Several methodological limitations related to restraint and seating conditions should be considered. Detailed quantitative measurements of head restraint height, head-to-restraint distance, seatback angle, and precise occupant posture were not instrumented or mechanically standardized. In addition, although participants were instructed not to brace for impact, anticipatory muscle activation could not be completely excluded. These factors are known to influence cervical spine kinematics and neuromuscular responses during low-speed rear-end collisions and may have affected the observed results. However, these conditions were intentionally maintained within typical real-world ranges to preserve ecological validity, as the primary aim of this study was not to isolate individual restraint parameters but to evaluate whether objectively detectable structural or electrophysiological cervical spine injuries occur under everyday low-speed rear-end collision conditions.

Pain assessment in this study was qualitative rather than quantitative. Only the presence and anatomical distribution of pain were recorded, without the use of standardized pain intensity scales such as the Visual Analogue Scale. Consequently, changes in pain severity over time could not be statistically analyzed. Nevertheless, pain assessment was included as a supplementary clinical observation rather than a primary outcome, as the main focus of this study was objective structural and electrophysiological evaluation. Despite these limitations, conducting controlled low-speed collision experiments in human volunteers involves substantial ethical and safety constraints, which inherently restrict sample size and participant diversity. Nonetheless, this study provides valuable objective data by integrating kinematic, radiological, and electrophysiological assessments. Future studies incorporating larger cohorts, comprehensive demographic profiling, standardized seat configurations, and inclusion of vulnerable populations are warranted to further validate and extend these findings.

Although the present findings may have potential clinical and insurance-related implications, these interpretations should be strictly confined to the experimental conditions of this study. Specifically, the results apply to healthy adult volunteers exposed to controlled and anticipated low-speed rear-end collisions, using specific vehicles and seating configurations, with short-term follow-up. Real-world collisions often involve unexpected impacts, heterogeneous occupant characteristics, variable restraint systems, and longer-term symptom evolution, which were not captured in this study. Therefore, caution is warranted when extrapolating these findings to broader clinical decision-making or insurance assessments.

## 5 Conclusion

In this study, we aimed to assess the occurrence of cervical spine injuries following low-speed rear-end collisions by comparing data from vehicle impacts with those from bumper car collisions and everyday driving-induced shocks. MRI comparisons before and after low-speed collision tests showed no remarkable changes, indicating no structural damage to the cervical spine. Additionally, nerve conduction studies (CMAP and SNAP) and electromyography results were interpreted as normal with no abnormal spontaneous activities, confirming the absence of electrophysiological abnormalities. Based on expert interpretations by radiology and rehabilitation medicine specialists, the present findings indicate that, in healthy adults exposed to controlled and anticipated low-speed rear-end collisions with a ΔV of up to 8 km/h, no objectively detectable structural or electrophysiological cervical spine abnormalities were identified under the tested conditions. However, these findings are limited to the specific experimental conditions of this study, including the study population, vehicle and seating configurations, and short-term follow-up. Further studies are necessary to evaluate long-term effects, real-world unanticipated collisions, and outcomes in vulnerable populations, as well as to develop strategies for minimizing pain and discomfort following low-speed impacts.

## Practitioner summary

*This study evaluated cervical spine injury risks in low-speed rear-end collisions using kinematics, MRI, and neurophysiological tests. Findings showed no structural or electrophysiological abnormalities at impact velocities up to 8 km/h, suggesting negligible injury risk. Results provide evidence-based guidance for clinical assessment and insurance-related decision-making in minor collisions.*

## Data Availability

The data that support the findings of this study are available from the corresponding author upon reasonable request.
